# Association between Response to Albendazole Treatment and β-Tubulin Genotype Frequencies in Soil-transmitted Helminths

**DOI:** 10.1371/journal.pntd.0002247

**Published:** 2013-05-30

**Authors:** Aïssatou Diawara, Carli M. Halpenny, Thomas S. Churcher, Charles Mwandawiro, Jimmy Kihara, Ray M. Kaplan, Thomas G. Streit, Youssef Idaghdour, Marilyn E. Scott, Maria-Gloria Basáñez, Roger K. Prichard

**Affiliations:** 1 Institute of Parasitology, McGill University, Sainte-Anne-de-Bellevue, Quebéc, Canada; 2 Department of Infectious Disease Epidemiology, School of Public Health, Faculty of Medicine, St Mary's Campus Imperial College London, London, United Kingdom; 3 Eastern and Southern Africa Center of International Parasite Control, Kenya Medical Research Institute, Nairobi, Kenya; 4 Department of Infectious Diseases, College of Veterinary Medicine, University of Georgia, Athens, Georgia, United States of America; 5 Department of Biological Sciences, University of Notre Dame, Notre Dame, Indiana, United States of America; 6 Sainte Justine Research Centre, Université de Montréal, Montréal, Québec, Canada; Swiss Tropical and Public Health Institute, Switzerland

## Abstract

**Background:**

Albendazole (ABZ), a benzimidazole (BZ) anthelmintic (AH), is commonly used for treatment of soil-transmitted helminths (STHs). Its regular use increases the possibility that BZ resistance may develop, which, in veterinary nematodes is caused by single nucleotide polymorphisms (SNPs) in the β-tubulin gene at positions 200, 167 or 198. The relative importance of these SNPs varies among the different parasitic nematodes of animals studied to date, and it is currently unknown whether any of these are influencing BZ efficacy against STHs in humans. We assessed ABZ efficacy and SNP frequencies before and after treatment of *Ascaris lumbricoides*, *Trichuris trichiura* and hookworm infections.

**Methods:**

Studies were performed in Haiti, Kenya, and Panama. Stool samples were examined prior to ABZ treatment and two weeks (Haiti), one week (Kenya) and three weeks (Panama) after treatment to determine egg reduction rate (ERR). Eggs were genotyped and frequencies of each SNP assessed.

**Findings:**

In *T. trichiura*, polymorphism was detected at codon 200. Following treatment, there was a significant increase, from 3.1% to 55.3%, of homozygous resistance-type in Haiti, and from 51.3% to 67.8% in Kenya (ERRs were 49.7% and 10.1%, respectively). In *A. lumbricoides*, a SNP at position 167 was identified at high frequency, both before and after treatment, but ABZ efficacy remained high. In hookworms from Kenya we identified the resistance-associated SNP at position 200 at low frequency before and after treatment while ERR values indicated good drug efficacy.

**Conclusion:**

Albendazole was effective for *A. lumbricoides* and hookworms. However, ABZ exerts a selection pressure on the β-tubulin gene at position 200 in *T. trichiura*, possibly explaining only moderate ABZ efficacy against this parasite. In *A. lumbricoides*, the codon 167 polymorphism seemed not to affect drug efficacy whilst the polymorphism at codon 200 in hookworms was at such low frequency that conclusions cannot be drawn.

## Author Summary

The soil-transmitted helminths (STH) *Ascaris lumbricoides*, *Trichuris trichiura* and the hookworms *Ancylostoma duodenale* and *Necator americanus* are endemic in many tropical countries. Regular treatment with albendazole or mebendazole is the major means for controlling STHs. However, repeated treatment with the same class of benzimidazole anthelmintics has caused resistance in veterinary parasites, characterized by mutations at either codon 200, 167 or 198 in the β-tubulin gene. There is a concern that resistance may develop in human STH. Drug efficacy and mutation frequencies were assessed in *T. trichiura*, *A. lumbricoides* and hookworms collected in Haiti, Kenya and Panama prior to and after albendazole treatment. In *T. trichiura* from Haiti and Kenya, a significant increase of the frequency of the mutation at codon 200 was identified after treatment and drug efficacy was mediocre. Against *A. lumbricoides*, albendazole efficacy was good, even though the frequency of a mutation at codon 167 was relatively high, suggesting that, in this nematode, the codon 167 polymorphism does not impact efficacy. In hookworms, the mutation at codon 200 was identified, but at low frequencies and the response to albendazole was good. We conclude that monitoring for possible resistance in control programmes should be undertaken.

## Introduction

*Ascaris lumbricoides* (roundworm), *Trichuris trichiura* (whipworm) and *Necator americanus/Ancylostoma duodenal*e (hookworms) are the most common species of soil-transmitted helminths infecting humans worldwide. More than a billion people are infected with at least one species and 300 million are estimated to have severe infections with more than one of these parasites [1]. These infections are endemic in tropical and sub-tropical regions of the developing world and are associated with poverty, lack of clean water, and poor sanitation [2]. School-age children are the most at risk of infection with STHs and early childhood infections contribute significantly to debilitation [3]. Infected children can be malnourished and experience stunting growth and intellectual retardation, with cognitive and educational deficits [1]. Because of all these characteristics and according to estimates by the World Health Organization (WHO) [4], STHs are included in the group of the so-called neglected tropical diseases (NTDs). The main intervention to control STH infections at a community level is based on periodic mass drug administration (MDA) of the benzimidazole (BZ) anthelmintic (AH) drugs, albendazole (ABZ) or mebendazole (MBZ) [5], that reduce the prevalence and intensity of infections [6]. However large-scale chemotherapy programmes with these drugs have the potential to exert selection pressures on the causing parasites, which may favour the development of drug resistance. Recently, expansion of MDA programmes for STHs have highlighted the need to monitor for the possibility that resistance may develop [7], [8]. This development would have important adverse consequences on the benefits provided by deworming programmes [9], [10]. BZ resistance in other parasitic nematodes is caused by a single nucleotide polymorphism (SNP) in the β-tubulin gene at codon positions 200 (T→A), 167 (T→A), or 198 (A→C) [11]–[13]. The frequency and relative importance of these different SNPs varies among the nematode species studied to date [13]–[15]. Molecular markers have been developed to identify SNPs in *A. lumbricoides*, *T. trichiura* and hookworms and wild-type and mutant-type control plasmids have been constructed to obtain the genotype profiles of “susceptible-type” parasites (which do not have mutations at position 200, 167 and 198) and “mutant-type” parasites (that contain mutations in one of these three positions) [16]. The codon 200 polymorphism has been identified in *T. trichiura* populations collected from untreated subjects in Kenya and from treated subjects in Panama [17], and in hookworms collected in Haiti in an area periodically treated with ABZ [16]. In the present report, our aim was to investigate the field efficacy of ABZ against STH infections in countries where polymorphism in β-tubulin had previously been identified, and to assess the frequency of each SNP prior to, and after ABZ treatment within each country and for each of the three main nematode species.

## Materials and Methods

### Study Design and Study Areas in Haiti, Kenya and Panama

**Figure 1:**
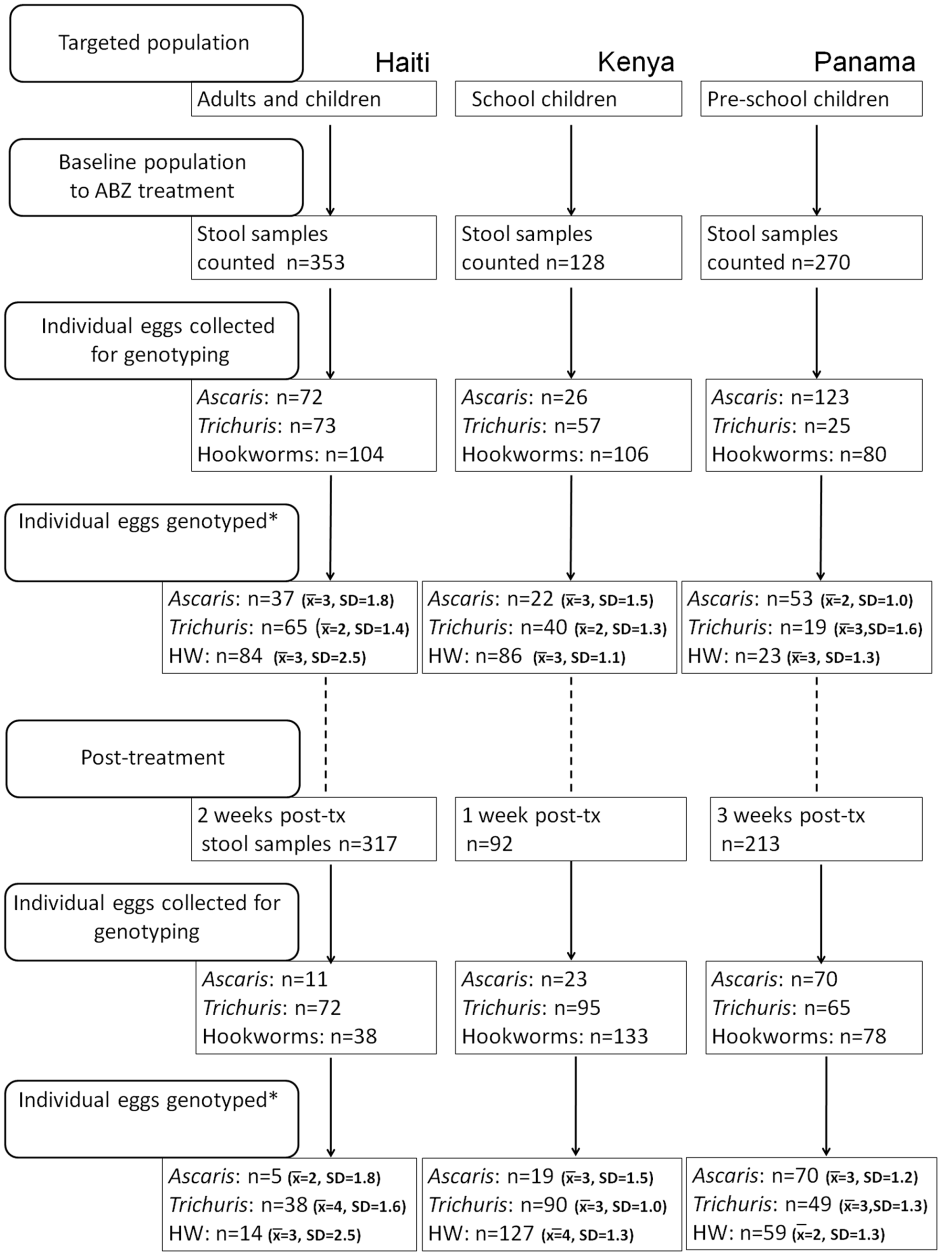
Flow chart of the study designs in Haiti, Kenya and Panama. *The number of eggs genotyped is different from the number of eggs collected. This is due to failures in DNA extraction, PCR amplification or Pyrosequencing. x̄ represents the mean number of eggs sampled per host; SD = standard deviation. The dashed lines mean that the connection between the two boxes is not direct.

Three cross-sectional studies were carried out between 2008 and 2010 in three countries located in different geographical areas, including Haiti in the Caribbean, Kenya in East Africa and Panama in Central America. These studies were integrated into the following control and evaluation programmes of ABZ use: a national health programme to fight lymphatic filariasis (LF) and intestinal worms in Haiti (in collaboration with the Hôpital Ste. Croix and the Centers for Disease Control (CDC)); school health and nutrition programmes in Kenya, and health and nutrition programmes in Panama. Thus, each study included a treatment with ABZ, stool examinations, and genotyping of the β-tubulin gene in eggs collected before and after ABZ treatment to assess the drug efficacy against STHs and to examine at each time point the frequency of possible SNPs associated with ABZ resistance in nematodes of veterinary importance. The study designs in Kenya, Panama and Haiti (Figure 1) were different because each study was part of a separate national and local MDA programme. Thus, different protocols were applied rather than a purposely designed multi-study site single protocol.

### Study in Haiti

The study in Haiti included stool collections prior to, and after a drug treatment given in 2009. In Haiti, the study sites were located in the West and Southeast Haitian departments known to be naive for MDA with ABZ. Individuals from five endemic communities who reached the inclusion criterion (older than two years old) were randomly selected. All potential participants were informed by a trained community leader of the purpose and methods of the study and gave their oral consent (in the case of children, consent was obtained from a parent). A total of 353 stool samples were collected and analyzed prior to ABZ treatment. Treatment was then distributed to all people of each community (400 mg ABZ and 6 mg/kg diethylcarbamazine (DEC)). ABZ was supplied as a donation from GlaxoSmithKline (GSK). Participants in the study were not observed during the treatment administration. However, the compliance to treatment was evaluated post-treatment by a questionnaire and relied on self-reported information of each participant, and on pre and post-treatment egg counts. Samples from participants who were not treated with ABZ were not included in the analysis. Follow-up faecal samples (n = 317) were collected two weeks after the drug treatment.

### Study in Kenya

School-aged children from two schools located in Kwale District were enrolled in the study. Although the schools were selected because at the time of the study they were not involved in any other STH deworming programmes, pupils had received ABZ and DEC six month prior to our study for LF treatment. Stool samples were collected prior to ABZ treatment in both schools (n = 128) and then all children received 400 mg ABZ under the supervision of the school teachers. The drug was provided by the Kenyan Ministry of Health and was manufactured by GSK. Seven days later, a follow-up collection was undertaken (n = 92). In one of the schools, the stool collections before and after ABZ treatment were done over two consecutive days and the results averaged.

### Study in Panama

The field study was conducted in the region of Comarca Ngäbe-Buglé [18]. Stool samples were collected from pre-school children at different time points over a period of 16 months from July 2008 to October 2009. Two treatments with ABZ, as a suspension (200 mg: one to two years of age; 400 mg: three to five years of age) were distributed, once in 2008 and the second. nine months later in 2009 [19]. The drug was provided by the Panamanian Ministry of Health. In the present study, we only analyzed the faecal samples collected prior to treatment and three weeks after treatment in 2009 since the baseline prevalence for STHs was very low in the 2008 samples. Prior to this second ABZ treatment, stool samples were collected from 270 pre-school aged children and follow-up samples were available from 222 children. Children were observed while received the ABZ treatment, and only samples from treated children were included in the analysis.

### Ethics Statement

In the three studies, all instances of consent were informed. Ethical approval for Haiti was obtained from the Institutional Review Board of the Centers for Diseases Control and Prevention, Atlanta, Georgia, US (Dr. Patrick Lammie), and the Ethics Committee of the Hôpital Ste Croix, Haiti and included the collection of stool samples, examination of stool samples for helminth eggs and DNA analysis of helminth eggs.

Informed consent was obtained from all adult participants and from parents or legal guardians of all minors. A parent or legal guardian gave consent in every case of child participation. Based on past experience, it was likely that some people in the communities would not be able to read. A waiver of written informed consent on the basis that the research presented no more than minimal risk of harm to the subjects and involved no procedures for which written consent is normally required outside the research context in this setting, was requested and approved. The use of oral consent was previously approved by the IRB. Subjects were offered a written copy of the IRB approved consent form. The contents of the approved consent form were explained to each household, following which verbal consent was obtained. The reader of the consent form and a witness signed a copy of the form to indicate the subject's agreement.

The study in Kenya was approved by the Kenya Medical Research Institute (KEMRI) Ethical Review Committee. The informed consent process, which was approved by the KEMRI IRB specifically stated that the study was included in the national programme on surveillance of disease control and followed established government procedure. The school heads organized meetings with their parent teacher association to obtain agreement for the project. An information sheet was provided. It was emphasized that the participation in the study by children was voluntary and that they may refuse to participate. All children were treated with ABZ and praziquantel in the study. Finally, all samples were anonymised. As part of a national control programme, informed consent was not documented for each individual. The content of the study was verbally explained in detail to parents/guardians, teachers and children from each school. It was done orally as it was necessary to have this explained in local languages. The use of oral consent was previously approved by the IRB. A parent or legal guardian gave oral informed consent in every case of child participation.

The study in Panama was approved by the McGill University Review Board in Canada, the Instituto Conmemorativo de Gorgas and local indigenous leaders in Panama. Written informed consent was obtained from primary caregivers for their own participation as well as that of their children. They were provided with an explanation of the study, its significance, and of participant requirements and rights. They were given an opportunity to ask questions in Spanish and in the local language. A parent or legal guardian gave consent in every case of child participation.

### Stool Sample Procedure and Examination

In all studies and for each intervention (before and after ABZ treatment), labelled containers were distributed to each participant and collected the following morning from the community leader in Haiti, schools in Kenya, and each participant's home in Panama. Three diagnostic techniques: McMaster, Kato-Katz, and FLOTAC were used to identify STH eggs and determine the number of egg per gram (epg) in the faecal material collected before and after ABZ treatment.

In Haiti we used a modified McMaster technique on each collected sample. One gram of faeces was suspended with water and the solution was stirred until it was completely broken apart. The mixture was poured through surgical gauze into a centrifuge tube. After centrifugation for 10 min at 15,000 rpm, the supernatant was poured off and the tube containing the sediment was filled with saturated sucrose solution and then gently stirred. After 10 min, an aliquot of the flotation fluid from the upper surface of the solution was transferred into each compartment of a McMaster chamber. The eggs were counted in both chambers using a low power objective (×10). The number of epg of faeces was obtained by multiplying the total number of eggs counted in the two chambers by 50 [20].

In Kenya, the McMaster (as described above) and Kato-Katz techniques [21] were used on samples from one school and Kato-Katz alone was used in the other school. For Kato-Katz, the number of eggs counted was multiplied by 24 to obtain the epg [21].

In Panama, the identification of eggs and the assessment of epg were performed using Kato-Katz [21] and FLOTAC [22], [23], as previously described [19].

One of the primary objectives of the study was to assess the frequency of SNPs associated with resistance to ABZ (seen in the veterinary nematode *Haemonchus contortus*) in the β-tubulin gene of STHs collected from untreated and treated subjects. Thus, from the three studies, eggs from positive subjects were recovered using a saturated sucrose solution with centrifugation, and recovered eggs were preserved in 70% alcohol until use for molecular analysis.

### Examination of the β-tubulin Gene and Assessment of SNPs

#### DNA Isolation from Individual Eggs of STHs

Eggs previously preserved in 70% alcohol were washed in distilled water and separated by species under a dissecting microscope. Individual eggs were isolated with a 10 µl pipette and then placed into a PCR tube. Genomic DNA of each individual egg was then extracted according to a protocol elaborated by Lake and colleagues [24] and adapted to STHs. One hundred millilitres of lysis buffer (50 mM KCl, 10 mM Tris pH 8.3, 2.5 mM MgCl_2_, 0.45% Nodidet P-40, 0.45% Tween 20, 0.01% gelatine) was previously prepared, and an aliquot of 1 ml of this lysis buffer was taken to which 10 µl proteinase K (10 mg/ml) and 10 µl β-mercaptoethanol (Sigma-Aldrich, St Louis, MO, USA) were added. The solution of lysis buffer with proteinase K and β-mercaptoethanol was placed at −20°C for one week. Individual eggs were placed into a labelled PCR tube and 15 µl of the mixture was added. The tube was subsequently frozen at −80°C for 30 min and then incubated at 60°C for 2 h. This procedure of freezing and heating the egg solution helps the digestion of the eggshell to facilitate DNA extraction. The proteinase K was inactivated by heating to 94°C for 15 min.

#### Genotyping of β-tubulin Gene Positions 167, 198 and 200 in *A. lumbricoides*, *T. trichiura* and Hookworms

After DNA extraction, all samples were subjected to PCR to amplify small fragments surrounding the SNP at codon positions 167, 198, and 200 in the β-tubulin genes of *A. lumbricoides* (FJ501301.1, GenBank acc. number), *T. trichiura* (AF034219.1 GenBank acc. number) and hookworms (EF392851.1 GenBank acc. number). All primers used for genotyping with the Pyrosequencer were designed with the PyroMark Assay Design Software (Qiagen, version 2.0) to amplify single egg DNA. To allow for nested PCRs on single eggs, some primers were different from those used in our previous study [17] in which the DNA concentrations were higher. For PCR, 2 µl of lysate was used as template in a 20 µl reaction containing 2 µl 10×PCR buffer, 1 µl [50 mM] MgSO_4_, 1 unit Platinum Taq DNA Polymerase High Fidelity, 1 µl of sense and antisense primers [10 µM] (details in Supplementary Table S1) 1 µl dNTP mix [10 µM], and distilled water to 20 µl. PCR reactions were performed with the following cycling parameters: 94°C for 3 min followed by 30 cycles of 94°C, 59°C for 45 s and 68°C for 1 min with a final extension step at 68°C for 5 min. A second PCR reaction using the earlier PCR product as template was necessary to visualize the product on an agarose gel. The reaction contained 5 µl 10×PCR buffer, 2 µl [50 mM] MgSO_4_, 1 unit Platinum Taq DNA Polymerase High Fidelity, 1 µl of sense and antisense primers [10 µM], 1 µl dNTP mix [10 µM], and distilled water to give a final volume of 50 µl. The same primers were used for each species amplified except for the antisense primer for codon position 198–200 of *A. lumbricoides* (5′ CAGATGTCGTACAAAGCCTCATT 3′, position). For genotyping, all antisense primers were biotinylated at their 5′end as described [17]. The amplification conditions were 94°C for 3 min followed by 40 cycles of 94°C, 59°C for 45 s and 68°C for 1 min with a final extension step at 68°C for 5 min. Sequencing primers for SNP analysis were used to genotype all PCR products at the codon positions 167, 198 and 200 in a Pyrosequencer (Biotage AB, Charlottesville, VA, USA) (Supplementary Table S1). Parasites genotyped did not necessary come from the same subjects sampled before and after treatment. Eggs were extracted from as many hosts as possible, though finding sufficient number post-treatment was restricted by low egg density. Therefore all eggs, from the same community or school, were pooled, before analysis of genotypes of individual eggs. The number of eggs isolated and genotyped are presented in the flow-chart (Figure 1).

### Statistical Analysis

The treatment efficacy on *A. lumbricoides*, *T. trichiura* and hookworms was evaluated by the egg reduction rate (ERR) for each diagnostic method applied in each country: McMaster (in Haiti, and Kenya), Kato-Katz (In Kenya and Panama) and FLOTAC (in Panama). The ERR was calculated, at the group level, as the ratio of the difference between the arithmetic mean of the pre- and post-treatment faecal egg count (FEC) to the pre-treatment arithmetic mean, expressed as a percentage, i.e. ignoring individual variability [25]. The negative individuals at baseline were still sampled at the post-treatment collection and the resulting data were included for the calculation of ERR. Uninfected subjects were included in the mean of the FEC. Confidence intervals of each ERR estimate were determined using a bootstrap resampling method (with replacement) over 10,000 replicates in R (version 2.15.0, Vienna Austria, http://www.R-project.org).

Genotype frequencies of SNPs at positions 167, 198 and 200 in *A. lumbricoides*, *T. trichiura* and hookworms obtained at the pre-treatment collections were compared with the genotype frequencies of the same SNPs obtained at the post-treatment collections using Fisher's exact test within GraphPad Prism (GraphPad software, San Diego, CA, USA).

Deviation from Hardy-Weinberg equilibrium (HWE) was analyzed for the β-tubulin gene at position 200 in *T. trichiura* using Arlequin version 3.1 software [26], where the p-value was calculated based on the Markov-chain method [27]. Deviations from the HWE were not determined for *A. lumbricoides* or hookworm for reasons explained below. When a departure from HWE was observed in *T. trichiura*, we estimated the maximum likelihood frequency of a null allele at position 200. This estimate was calculated using an Expectation-Maximization Algorithm of Dempster and colleagues [28] (EM Algorithm, http://132.206.161.123/em.html).

## Results

### Genotype Frequencies for the β-tubulin Gene in *T. trichiura*

In *T. trichiura*, the three codon positions 167, 198, and 200 were found to be polymorphic in samples collected from untreated and treated subjects in Haiti, Kenya, and Panama.

**Figure 2:**
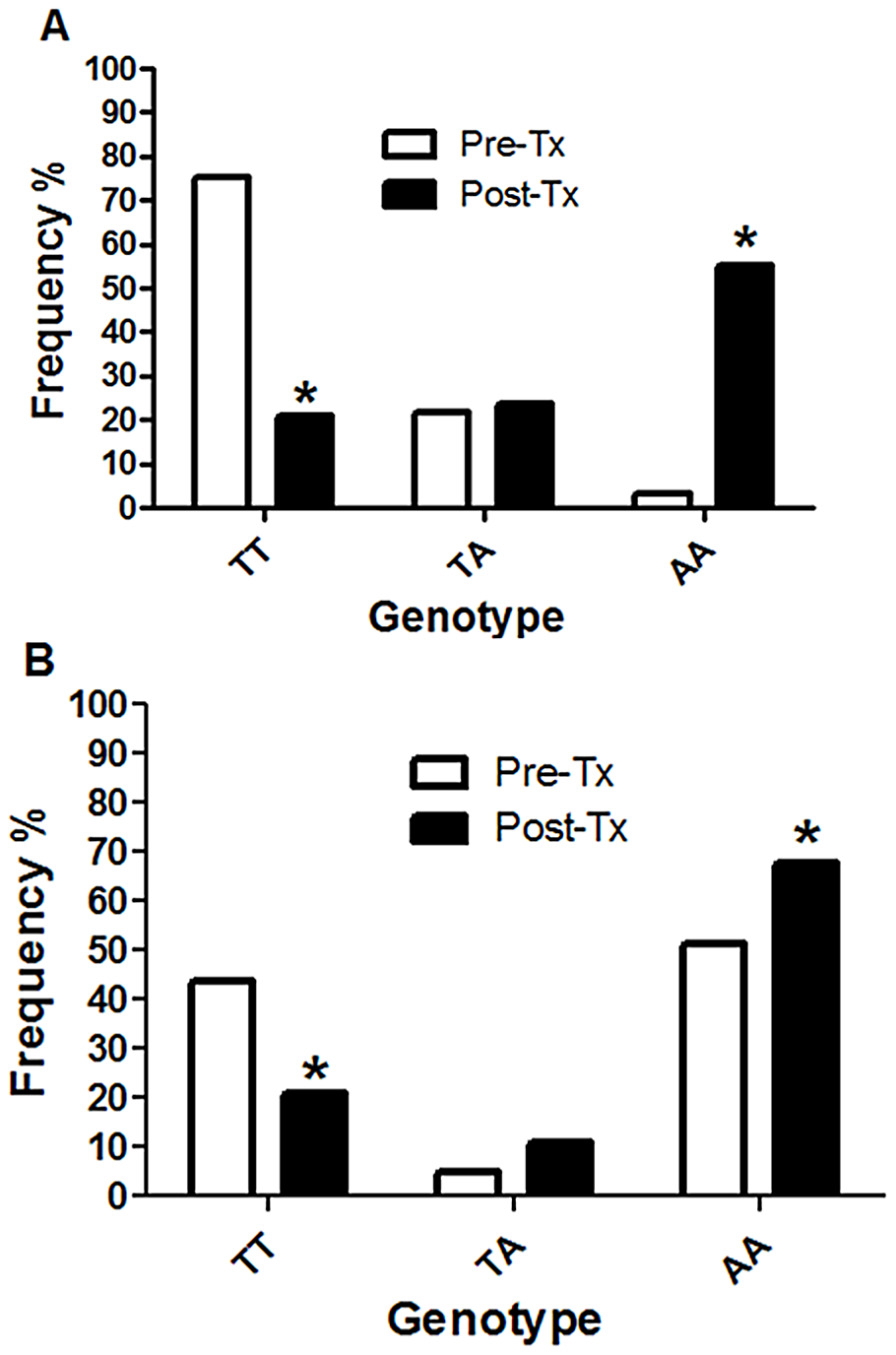
Genotype frequencies of the β-tubulin gene position 200 in *T. trichiura* from Haiti and Kenya. Genotype frequencies of *T. trichura* collected in Haiti (A) and in Kenya (B); Number of individual *T. trichiura* eggs genotyped according to the available material, in Haiti was 65 in the untreated group (pre-Tx) and 38 in the treated group (post-Tx), in Kenya was 40 in the untreated group and 90 in the treated group. Sequences were diploid, TT indicates the homozygous susceptible-type TTC/TTC, TA the heterozygous TTC/TAC and AA, the homozygous resistance-type TAC/TAC; Tx = treatment, *Indicates a significant difference (p<0.001) in genotype frequency between the pre- and post treatment groups. P-values were obtained by Fisher's exact test.

**Figure 3:**
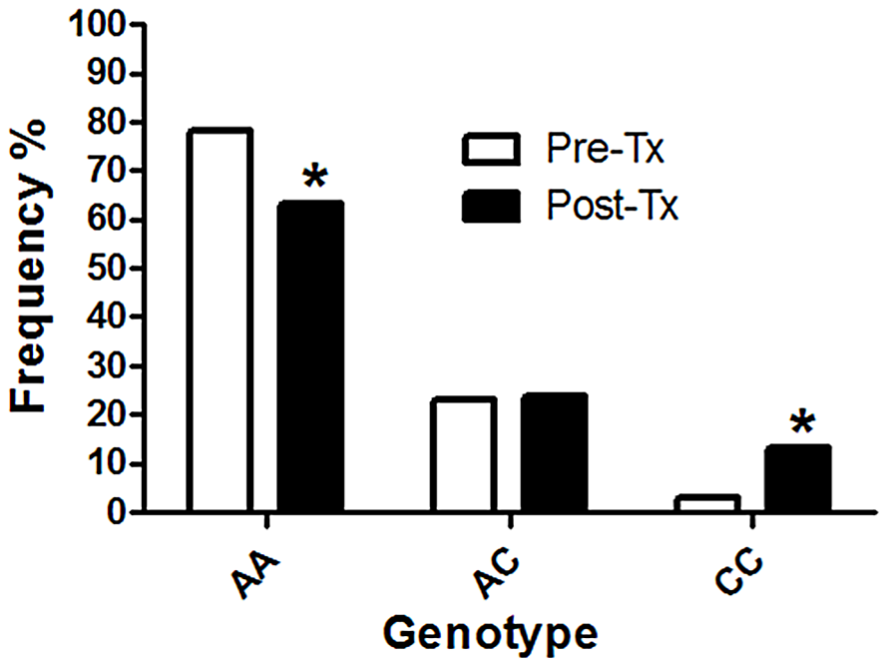
Genotype frequencies of the β-tubulin gene position 198 in *T. trichiura* from Haiti before and after ABZ treatment. Genotype frequencies of *T. trichura* collected in Haiti. The number of individual *T. trichiura* eggs genotyped, according to the available material, was 65 in the untreated group (pre-Tx) and 38 in the treated group (post-Tx). Sequences were diploid, AA indicates the homozygous susceptible-type GAA/GAA, AC the heterozygous GAA/GCA and CC, the homozygous resistance-type GCA/GCA; Tx = treatment, *Indicates a significant difference (p<0.001) in genotype frequency between the pre- and post treatment groups. P-values were obtained by Fisher's exact test.

In Haiti we analyzed 65 individual *T. trichiura* eggs from 30 untreated subjects and 38 from 14 treated subjects. We recorded 11% and 47% experimental failure (includes DNA extraction, PCR amplification and Pyrosequencing) in pre- and post-treatment samples, respectively. Before treatment the T→A SNP at codon position 200 (SNP200) was identified at low frequency; 3.1% of individual eggs genotyped were homozygous resistance-type (AA) and 23.1% were heterozygous (TA). After treatment, there was a significant increase in the frequency of the homozygous resistance-type, from 3.1% to 55.3% (p<0.001), and a statistically significant decrease of the homozygous susceptible-type, from 75.4% to 21.1% (p<0.0001) (Figure 2A). The A→C SNP at codon position 198 was also found and showed a statistically significant change in frequency following treatment. However, the changes at codon 198 were less pronounced than at codon 200. After treatment, there was a significant increase of homozygous resistant-type from 3.1% to 13.2% (p<0.001), and a significant decrease of homozygous susceptible-type from 73.8% to 63.2% (Figure 3).

In Kenya, 40 individual *T. trichiura* eggs from 20 untreated subjects and 90 eggs from 31 treated subjects were genotyped. We recorded 30% and 5% experimental failure in pre- and post-treatment samples, respectively. Only the codon 200 SNP was polymorphic. The same trend observed in Haiti was seen with a statistically significant increase, from 51.3% to 68.5% (p = 0.019) of homozygous resistance-type, and a significant decrease, from 48.6% to 21.4% (p = 0.019) of homozygous susceptible-type after treatment (Figure 2B).

In Panama we genotyped 19 “pre-treatment” *T. trichiura* eggs from 10 subjects, which were collected 9 months after an earlier treatment, and 49 post-treatment eggs from 21 subjects collected 3 weeks after this second ABZ treatment. We observed homozygous resistance-type (78.9%) and homozygous susceptible-type (21.1%), at codon 167, in the pre-treatment collection. For codons 198 and 200, 84% of the pre-treatment *T. trichiura* egg samples failed the PCR amplification and therefore it was not possible to assess the genotype frequencies for these positions. At the post-treatment collection, the codon 167 polymorphism was still present in the treatment survivors as homozygote resistance-type in 16.3% of individual eggs genotyped. For codons 198 and 200, we observed a predominance of homozygous susceptible-type genotypes, 97.6% and 88.1% respectively; and a low frequency of heterozygous, 2.4% (for each position) and homozygous resistance-type, 9.5% (for codon 200).

### Genotype Frequencies of β-tubulin Gene Positions 167, 198 and 200 in *A. lumbricoides*

In *A. lumbricoides*, the codon position 167 of the β-tubulin gene was polymorphic in parasites collected from untreated and treated subjects in Haiti, Kenya and Panama, whereas the codon positions 198 and 200 were monomorphic. The SNP at position 167 identified was confirmed by conventional Sanger sequencing and real time PCR (data not shown).

In Haiti, we genotyped 37 individual *A. lumbricoides* eggs from 13 untreated subjects and five eggs from three treated subjects. We recorded 49% and 28% experimental failure in pre- and post-treatment samples. Prior to treatment, homozygous resistance-type (40%) and heterozygous (60%), at codon 167, were present in the population; however, only heterozygotes were detected after treatment.

In Kenya, 22 individual *A. lumbricoides* eggs from 6 untreated subjects and 19 eggs from 4 treated subjects were genotyped. We recorded 15% and 17% experimental failure in pre- and post-treatment samples, respectively. The predominant genotype frequency identified was the homozygous resistance-type (72.7%) at codon 167. After treatment, there was no significant difference in the homozygous resistance-type frequency; however, as observed in *A. lumbricoides* collected in Haiti, at codon 167 there was a statistically significant increase of heterozygotes from 4.5% to 21.1% (p<0.001), and also a significant decrease of homozygous susceptible-type from 22.7% to 5.3% (p<0.001).

In Panama, 53 individual eggs were genotyped from 28 untreated subjects and 70 eggs from 20 treated subjects. We recorded 57% of experimental failure only in pre-treatment samples. As seen previously in Haiti and Kenya, the most abundant genotype at codon 167 was homozygous resistance-type (97.7%). In this population the lowest genotype frequency was identified as homozygous susceptible-type (2.3%). After treatment, the percentage of both genotypes did not change significantly (96.7% and 3.2%, respectively) (Table 1).

**Table 1:** Genotype frequencies of β-tubulin position 167 in *A. lumbricoides* before and after ABZ treatment.

	Frequency (%)					
	Haiti		Kenya		Panama	
Genotypes	Pre-Tx^4^ (n = 37)	Post-Tx (n = 5)	Pre-Tx (n = 22)	Post-Tx (n = 19)	Pre-Tx (n = 53)	Post-Tx (n = 70)
**TT** 1			22.7^a^	5.3^a^		
**TA** 2	60	100.0	4.5^b^	21.1^b^	2.3	3.2
**AA** 3	40		72.7	73.7	97.7	96.8

1 TT = homozygous susceptible-type TTC/TTC.

2 TA = heterozygous TTC/TAC.

3 AA = homozygous resistance-type type TAC/TAC.

4 Tx = treatment.

The numbers in parentheses indicate the number of individual eggs genotyped. The letters (a,b) indicate significant difference (p<0.001) between the genotypes of the pre- and post treatment groups.

P-values were obtained from Fisher's exact test.

### Genotype Frequencies of β-tubulin Gene Positions 167, 198 and 200 in Hookworms

In hookworms, the codons 167 and 198 of the β-tubulin gene were monomorphic in all samples genotyped from Haiti, Kenya and Panama. Codon 200 polymorphism (TAC) was detected in 2 eggs collected in Kenya. In Kenya, 86 individual eggs from 28 untreated subjects and 127 eggs from 34 treated subjects were genotyped. We recorded 19% and 4% experimental failure in pre- and post-treatment samples, respectively. In the pre-treatment collection we identified homozygous resistance-type at low frequency (2.3%) and a predominance of homozygous susceptible-type (97.7%). After treatment the frequencies did not change significantly. In Haiti, we examined 84 hookworm eggs from 31 untreated subjects and 14 from five treated subjects and we did not identify any polymorphism at the SNP sites of interest. We recorded 19% and 63% of experimental failure for the samples from the pre- and post-treatment, respectively. In Panama, all 23 eggs analyzed from nine untreated subjects and 59 from 29 treated subjects were homozygous susceptible-type for all three positions. We recorded 71% and 24% of experimental failure for the samples from the pre- and post-treatment, respectively.

### Drug Efficacy

The arithmetic means of the faecal egg count (FEC) per gram are presented in Table 2. The standard error of the mean obtained shows a high variability of the FEC in the treated and untreated populations.

**Table 2:** Faecal egg count reduction rates for *A. lumbricoides*, *T. trichiura* and hookworms.

	Arithmetic mean of FEC^1^ at pre-treatment (95% CI^2^)			Arithmetic mean of FEC at post treatment (95% CI)			ERR^3^ (%) (95% CI)		
Country and Parasite	McMaster	Kato-Katz	FLOTAC	McMaster	Kato-Katz	FLOTAC	McMaster	Kato-Katz	FLOTAC
**Haiti, n**	353	4NA	NA	317	NA	NA			
*Ascaris*	370.0 (50.1–928.8)	NA	NA	1.6 (0–5.0)	NA	NA	99.9 (99.5–100)	NA	NA
*Trichuris*	48.4 (25.7–76.3)	NA	NA	24.5 (5.9–51.1)	NA	NA	49.7 (0–88.4)	NA	NA
HW^5^	209.1 (107.7–338.3)	NA	NA	2.9 (0.8–6.2)	NA	NA	98.6 (96.1–99.7)	NA	NA
**Kenya, n**	24	104	NA	42	92	NA			
*Ascaris*	2737.5 (12.5–8166.7)	330.5 (12.3–932.2)	NA	73.8 (0–221.4)	73.6 (0–195.1)	NA	97.3 (0–100)	80.3 (0–100)	NA
*Trichuris*	1650.0 (62.5–4750.0)	161.5 (69.2–282.1)	NA	217.1 (32.9–537.8)	145.2 (39.2–285.3)	NA	86.8 (0–98.7)	10.1 (0–78.1)	NA
HW	854.2 (64.6–2139.6)	279.6 (171.7–414.8)	NA	3.6 (0–9.5)	8.8 (2.3–17.5)	NA	89.9 (0–96.1)	96.8 (92.5–99.2)	NA
**Panama, n**	NA	92	108	NA	65	223			
*Ascaris*	NA	8346.2 (4914.9–12414.7)	1900.8 (1060.4–2840.2)	NA	3590.0 (451.3–7913.4)	171.9 (50.8–322.9)	NA	60.0 (0–95.1)	89.8 (75.8–97.3)
*Trichuris*	NA	280.8±170.1	40.2 (11.1–78.1)	NA	1.4 (0–3.5)	14.1 (5.2–25.3)	NA	99.5 (89.7–100)	65.1 (0–89.1)
HW	NA	192.5 (1.8–478.1)	72.6 (31.1–125.8)	NA	0.9 (0–2.3)	37.9 (8.3–90.4)	NA	99.8 (96.6–100)	47.8 (0–89.9)

1 FEC, faecal egg count, (egg per gram of stool).

2 CI, confidence interval. The confidence intervals of the egg reduction rate were calculated using a bootstrap resampling method [45].

3 ERR, egg reduction rate. The ERR estimates were obtained by dividing the difference between the arithmetic mean of the pre- and post-treatment FEC (at group level, ignoring individual variability) by the mean of the pre-treatment mean count (see text).

4 NA = Not applicable.

5 HW = Hookworm.

The ERR estimates calculated for *A. lumbricoides*, *T. trichiura* and hookworms for each diagnostic test applied in Haiti, Kenya and Panama are summarized in Table 2. The ERR from the same country estimated using two different diagnostic methods are not directly comparable as the number of samples tested (and therefore the hosts making up the ERR) were different. A direct comparison of the different diagnostic methods *per se* is beyond the scope of this paper and has been previously discussed elsewhere [29], [30]. In Haiti where only McMaster was applied, the ERR for *A. lumbricoides* was the highest (99.9% (95% CI 99.5–100.0)) followed by hookworms (98.6% (95% CI 96.1–99.7)) and *T. trichiura* (49.7% (95% CI 0.0–88.4)). In Kenya the ERR estimates differed between the Kato-Katz and McMaster methods. The highest ERR was obtained for hookworms, with Kato-Katz at 89.95% (95% CI, 0.0–96.1%) and McMaster at 96.8% (95% CI, 92.5–99.2%). This was followed by *A. lumbricoides* with 97.3% (95% CI, 0.0–100.0%) and 80.3% (95% CI, 0.0–100.0%), respectively, and finally by *T. trichiura* with 86.8% (95% CI, 0.0–98.7%) and 10.1% (95% CI, 0.0–78%). The ERR estimate based on Kato-Katz was the one considered as the sample sizes available were much higher (n = 104 and n = 92 at pre- and post-treatment, respectively) than those for McMaster (n = 24 and n = 42 at pre- and post-treatment, respectively). In Panama, the ERR estimates for FLOTAC were the ones considered as the number of samples analyzed was greater than those counted by Kato-Katz [19]. In Panama, the highest ERR estimate was for *A. lumbricoides*, at 89.8% (95% CI, 75.8–97.3%), followed by *T. trichiura* with 65.1% (95% CI, 0–89.1%), and finally by hookworms with 47.8% (95% CI, 0–89.9%).

### Hardy-Weinberg Equilibrium

Guo's Exact Hardy-Weinberg test [27] showed that there was a significant departure from Hardy-Weinberg expectations recorded in *T. trichiura* collected in Haiti (p = 0.0366) after treatment, and in Kenya before and after treatment (p<0.0001) for position 200 of the β-tubulin. This disequilibrium was characterized by a deficiency in the number of heterozygotes (Table 3). The estimated frequency of a null allele at position 200 showed no evidence (χ^2^ = 3.69) that a null allele was responsible of the paucity of heterozygotes. For positions, 167 and 198, respectively, polymorphism was either not found, or the differences between the frequencies of heterozygotes pre- and post-treatment were not significant.

**Table 3:** Observed (H_O_) and expected (H_E_) heterozygosity tested under HWE for SNP 200 in *T. trichiura*.

	Pre- treatment				Post- treatment			
	n^1^	H_O_	H_E_	p-value	n	H_O_	H_E_	p-value
**Haiti**	65	0.25	0.24	1.00	38	0.26	0.43	0.0366*
**Kenya**	40	0.15	0.50	<0·0001*	90	0.8	0.38	<0·0001*

1 n = sample size.

* Indicates a significant p-value; p-values were obtained from Guo's exact test.

## Discussion

One of the main findings of this study was the identification of a SNP at position 200 in *T. trichiura* samples collected in Haiti and Kenya at the pre-treatment collections. Thus, these findings suggest that the resistance-type allele (TAC_200_) already existed in these populations prior to ABZ treatment. This result was consistent with a previous report in which the codon 200 polymorphism was identified in adult *T. trichiura* from Kenya in an ABZ-naïve population [17]. This SNP was also present in human filarial nematodes from Burkina Faso, in samples obtained from pre-treatment patients at a moderate allele frequency (26.2%) [31].

However, it must be taken into consideration that ABZ is a commonly used AH in endemic countries, and yearly school-based helminth control programmes relying on ABZ and MBZ are also common in countries where our studies were performed [32]–[34]. Indeed, in Kenya, six months prior to our study, children from the selected schools had been treated with ABZ in the context of the LF control programme. In Panama, at the baseline collection (nine months earlier), the codon 200 polymorphism was identified (data not shown), and in a previous study carried out in the same region, the same SNP had been detected in *T. trichiura* [17]. Finally, in Haiti, in the context of the LF control programme, ABZ is widely distributed yearly in combination with DEC at the community level, in surrounding communities [32]. So, it may be possible that some participants in Panama or Haiti from the studied populations had travelled to treated areas or could have been infected with eggs from family members who had been previously treated with ABZ. Thus, it may be possible that parasites have been previously exposed to the drug and that some selection for the SNPs at codon position 200 had occurred prior to the beginning of MDA for STHs, or that in Kenya, selection at codon 200 may have developed following the previous rounds of ABZ treatment in the population.

Other findings were the identification of SNPs in *T. trichiura* at codon 167 in samples from Panama and at codon 198 in samples from Haiti and Kenya, prior to treatment. The identification of the same resistance-type allele at codon 198, in populations that are geographically separated suggests that this allele could be common in *T. trichiura* populations, even prior to ABZ treatment. However, the frequency of the resistance-type allele at codon 198, and the identification of the resistance-type allele at codon 167 in only one country suggest that these polymorphisms are less common than the codon 200 polymorphism. In the veterinary parasitic nematode *Haemonchus contortus*, it has been confirmed that the codon 200 polymorphism, which causes BZ resistance, is the predominant SNP associated with resistance, compared to the codon 167 polymorphism [13], or the codon 198 polymorphism [12], [15]. In addition, it was also interesting to note that the codon 167 polymorphism was always found alone and never associated with the SNPs at positions 198 and 200. This is consistent with observations made in *H. contortus* suggesting that the polymorphism at position 167 (TAC) does not occur in the same allele as the polymorphism at position 200 (TAC) or 198 (GCA) [12], [15].

The post-treatment examination of the resistance-type allele frequencies of *T. trichiura* collected in the three different geographical areas suggests that ABZ may be selectively eliminating worms carrying susceptible-type alleles and allowing worms with the resistance-type alleles to survive treatment. Indeed, in Haiti and Kenya one round of treatment significantly increased the frequency of the homozygous resistance-type at codon position 200 (TAC/TAC_200_) and some heterozygote eggs (TTC/TAC_200_) were also found after treatment. It was also found that the codon 198 polymorphism persisted after one treatment but at lower frequency compared to the codon 200 polymorphism. Thus, our results suggest that ABZ treatment could select for parasites harbouring the codon 200 (TAC) polymorphism. We tested whether the genotype frequencies of the β-tubulin gene at codon 200 obtained before and after treatment were in HWE. In Haiti, the increase of the homozygous resistance-type after treatment, for codon position 200, correlated with a departure from HWE illustrated by a paucity of heterozygotes and an excess of homozygotes. In Kenya, the β-tubulin at position 200 in *T. trichiura* was not in HWE before treatment as there was an excess of homozygotes. Different hypotheses may explain the HWE imbalance in genotype frequencies; one possible cause could be selection acting on the parasite populations.

The above data strongly suggest that the SNP at β-tubulin codon 200 may be responsible for the intermediate efficacy of ABZ against *T. trichiura*, where the ERR ranged from 10.1% (Kenya) to 65% (Panama). A poor efficacy of ABZ against *T. trichiura* has been repeatedly observed across a number of different studies [35]. In addition, it is not uncommon to find considerable variation of ERR estimates (ranging from 0 to 90%) in the literature [36], which, in addition to being explained by the use of different diagnostic methods (see above), is in agreement with the high resistance-type allele frequencies observed at loci 200 and 198 in Haiti and Kenya. If drug susceptibility is determined (in part) by these alleles, then AH resistance might develop relatively quickly in locations such as these. Resistance to BZ drugs is usually recessive [11] so high allele frequencies are required to generate the phenotypic diversity that leads to the poor drug efficacy that has been recorded.

Another finding of the study was the identification of the resistance-type SNP (based on comparison with studies in some veterinary nematodes), at codon position 167 in *A. lumbricoides* before and after treatment. In *A. lumbricoides* collected in Haiti, Kenya and Panama, the allele containing TAC at codon 167 was identified as homozygous at high frequency prior to ABZ treatment. The post-treatment genotype frequencies indicated that ABZ treatment did not change the frequency of the homozygous, resistance-type, codon 167 in Kenya and Panama, where sample sizes were similar, and heterozygotes remained in the population surviving treatment. It was also interesting to note that similar findings have been published for the veterinary parasitic nematode, *Teladorsagia circumcincta* [13]. In this case, it was demonstrated that BZ treatment did not change the frequency of the homozygous, resistance-associated codon 167 or that of heterozygotes. In a recent study, it was also found that *H. contortus* heterozygous at codon 167 were associated with susceptible phenotypes [15]. In contrast, in Haiti where the sample sizes were highly different between the pre- and post-treatment groups, we found that the resistance-type genotype frequency of *A. lumbricoides* at codon position 167 significantly increased after treatment, but the small sample size at the post-treatment collection made it difficult to draw clear conclusions.

The genetic data from *A. lumbricoides* suggest that the polymorphism at codon position 167 is common and may be naturally present. However, ERR estimates indicate that treatment was successful according to WHO standardized thresholds [37]. This implies that in *A. lumbricoides*, the mutation at codon position 167 may not have an impact on drug efficacy. In contrast, in intestinal trichostrongylid nematodes of livestock (e.g., *H. contortus*), the SNP 167 is rarely encountered but the homozygous allele containing TAC does confer BZ resistance in some species [38], [39]. It is also common in these species to have SNP associations between codons 167 and 200 that could confer high levels of BZ resistance [15].

It was interesting to find that in *A. lumbricoides*, all eggs genotyped were susceptible-types at codon positions 198 and 200. It is possible that should polymorphisms be found at codons 200 or 198 in *A. lumbricoides*, these polymorphisms may change the sensitivity of the parasite to the drug as has been described in veterinary parasites [12], [15]. It will be informative to investigate the β-tubulin gene of *A. lumbricoides* in areas where MDA has been implemented to determine whether the position 200 is ever polymorphic and whether it correlates with drug response.

Finally, the β-tubulin analysis in hookworms, at codon 200, 167 and 198, revealed the presence of a SNP at codon 200 in samples collected in Kenya at pre- and post-treatment. However, the frequency was very low and the ERR indicated a value consistent with ABZ treatment being successful. In other studies in which a reduced drug efficacy was detected polymorphism at codons 200 and 167 was not detected [40], [41].

The main limitation of the present study was the non homogeneity of the study designs in Haiti, Kenya and Panama. Indeed, the samples and data on response to treatment were obtained as part of different collaborations with ongoing treatment protocols rather than being designed specifically for our study. As such it was not possible to standardize the methods used for egg count estimations and exact time samples were taken after treatment. Furthermore, the studied populations were different in the three studies. In Panama and Kenya it was restricted to pre-school and school age children whereas in Haiti, adults and children were sampled. Furthermore, three diagnostic methods have been applied to quantify STHs. Resulting drug efficacies were variable within the same country, depending on the techniques and sample sizes. In another study, the McMaster and Kato-Katz gave similar results for the ERR [25]. It is important to mention that the sample sizes were different for the two diagnostic tests when performed in the same country. Our goal was not to compare test sensitivity for quantifying egg counts. The results reported here should be viewed as those arising from three different field treatment studies in which we used procedures to assess genotype and have compared the genotypes with assessments of ERR for each of the three separate treatment studies, observed with different methods and sampling schedules. As such the ERR results between the studies should not be compared; what was relevant for our investigations was the assessment of ERR by one or more conventional parasitological methods with the genotype determination for samples from the same communities.

The other limitation of the study was the low sample size of individual eggs genotyped due to a high percentage of PCR failure. This was caused by the difficulty in amplifying the DNA of individual eggs, possibly due to the presence of inhibitors from faeces that could prevent the DNA extraction [42]. Also, the low DNA concentration may have affected the success of the DNA amplification and pyrosequencing. In *T. trichiura* eggs from Panama, we recorded more DNA amplification failure for positions 198–200 than for position 167. One of the primers used to amplify the sequence around the codons 198 and 200 was in part in an intron. Polymorphism in this intron may have resulted in poor amplification in some samples. However, the samples that were successfully genotyped allowed identification of SNPs in *T. trichiura* which may be associated with the ability of worms to survive ABZ treatment. These results stress the importance of implementing, as integral part of MDA programmes, regular monitoring of drug efficacy and associated parasite genetics for prompt detection of drug resistance.

In this study, we estimated the ERR at the group level; it is a more accurate estimation of overall ERR because it can include negative individual values. The estimation of the ERR on a group basis by bootstrapping gave a range of [0–89%] in hookworms, whereas when estimated by Halpenny and colleagues [19] on individual basis the ERR was 89%.

Likewise, there is an urgent need to consider control strategies that will maintain a low resistance-type SNP prevalence and a high drug efficacy. Strategies have been described to maintain a high level of STH control while also delaying the possible development of drug resistance [7]. Drug combination has been used to combat the problem of AH resistance in veterinary parasites and human parasites [43], [44]. The pros and cons of various combinations should be carefully considered so that the chosen combination will have both a positive impact on drug efficacy and on a reduction of genetic selection for resistance.

### Conclusion and Future Directions

Comparable data on pre- and post-treatment SNP frequencies were obtained at the different study sites, despite differences in the study designs between sites. However, in the context of monitoring and surveillance of STH control programmes, it will be important to conduct multiple studies according to standardized multi-centre protocols in order to allow drug efficacies to be compared.

## Supporting Information

Table S1 Primers used for amplification of STHs β-tubulin SNP regions and for pyrosequencing.
(DOCX)
