# Supplementary material for: Association between Response to Albendazole Treatment and β-Tubulin Genotype Frequencies in Soil-transmitted Helminths
Source: PLoS Negl Trop Dis. 2013 May 30;7(5):e2247. doi: 10.1371/journal.pntd.0002247 (PMC3667785; doi:10.1371/journal.pntd.0002247)
Supplement: Table S1 — Primers used for amplification of STHs β-tubulin SNP regions and for pyrosequencing. (DOCX) [file pntd.0002247.s001.docx]

**Supplementary Table S1.**

| **Position 167** | | | |
| --- | --- | --- | --- |
| **STHs** | **Sense primer (5’3’)** | **Antisense primer (5’-3’)** | **Pyrosequencing primer** |
| ***A. lumbricoides*** | TCCGTGAAGAATACCCCGACA  (position 47-68) | GCCACACTTGAACCTGCTAACG  (position 54-75) | accccgacagaatcatgagctcg  (position 59-81) |
| ***T. trichiura*** | GAGTATCCTGACCGAATTATGACA  (position 1116-1139) | ACGACGTGAACAGTATCAAACAAC  (position 1171-1194) | TGACCGAATTATGACAACT  (Position 1124-1148) |
| **Hookworm** | GTGACTGTCTCCAGGTAATTCG  (position 867-888) | CTATAACGTACCTTTGGCGAGGG  (position 1065-1087) | GATAGAATCATGTCCTCGT  (Position 1035-1053) |
| **Position 198-200** | | | |
| **STHs** | **Sense primer (5’3’)** | **Antisense primer (5’-3’)** | **Pyrosequencing primer** |
| ***A. lumbricoides*** | agagccacagttggtttagatacg  (position 318-341) | AGGGTCCTGAAGCAGATGTC  (position 489-508)  CAGATGTCGTACAAAGCCTCATT  (position 476-498) | GGTTGAGAACACCGAT  (position 440-455) |
| ***T. trichiura*** | CGCCTTTTTAGGTTTCAGATACA  (position 1202-1224) | GTCTCCGTAAGTTGGTGTTGTTAA  (position 1339-1362) | GGTAGAGAACACGGACG  (Position 1266-1282) |
| **Hookworm** | TTTCCGACACTGTGGTTGAG  (position 1844-1863) | GAGTTCGTTACTAGCCAGCTCACC  (position 2006-2029) | GAGAATACAGATGAGACCT  (Position 1110-1128) |
